# Bodily-Contact Communication Medium Induces Relaxed Mode of Brain Activity While Increasing Its Dynamical Complexity: A Pilot Study

**DOI:** 10.3389/fpsyg.2018.01192

**Published:** 2018-07-09

**Authors:** Soheil Keshmiri, Hidenobu Sumioka, Junya Nakanishi, Hiroshi Ishiguro

**Affiliations:** ^1^Hiroshi Ishiguro Laboratories, Advanced Telecommunications Research Institute International, Kyoto, Japan; ^2^Graduate School of Engineering Science, Osaka University, Suita, Japan

**Keywords:** electroencephalography, multi-scale entropy, permutation entropy, differential entropy, cognition, touch, hug

## Abstract

We present the results of the analysis of the effect of a bodily-contact communication medium on the brain activity of the individuals during verbal communication. Our results suggest that the communicated content that is mediated through such a device induces a significant effect on electroencephalogram (EEG) time series of human subjects. Precisely, we find a significant reduction of overall power of the EEG signals of the individuals. This observation that is supported by the analysis of the permutation entropy (PE) of the EEG time series of brain activity of the participants suggests the positive effect of such a medium on the stress relief and the induced sense of relaxation. Additionally, multiscale entropy (MSE) analysis of our data implies that such a medium increases the level of complexity that is exhibited by EEG time series of our participants, thereby suggesting their sustained sense of involvement in their course of communication. These findings that are in accord with the results reported by cognitive neuroscience research suggests that the use of such a medium can be beneficial as a complementary step in treatment of developmental disorders, attentiveness of schoolchildren and early child development, as well as scenarios where intimate physical interaction over distance is desirable (e.g., distance-parenting).

## 1. Introduction

Touch establishes the first sensational channel for parent-infant physical bond and interaction (Gallace and Spence, 2010). In fact, the discovery of human C-tactile (CT) afferent unveils a preference for tactile information with socio-affective relevance (Löken et al., 2012). This, in part, explains the intensifying effect of pleasant touch on the emotional experience via other modalities (Knapp et al., 2012). For instance, the mere observation of touch in another human activates somatosensory cortex in healthy subjects (Blackmore et al., 2005). These findings help explain the pivotal role that interpersonal touch plays in human interactions and their physical and emotional well-being (Kutner et al., 2008; Gallace and Spence, 2010). Chatel-Goldman et al. (2014) report on the coupling of the electrodermal activity between interacting partners, regardless of the intensity, and the valence of their emotions, thereby enabling the emergence of a somatovisceral resonance between interacting individuals. In addition, a rich body of research provides evidence on the socioemotional significance of such interpersonal relationships and their positive physiological and biochemical effects (Field, 2010). Moreover, these observations are supported by the results of the study of the effect of the massage therapy on the physiological signals as well as EEG time series of brain activity of human subjects (Diego et al., 2004; Field et al., 2005; Field, 2010; Singh and et al., 2014).

Recent advances in intelligent robotics systems capable of communicating with human introduces a novel venue to further explore the interpersonal and societal interactions among people (Shibata, 2004; Haans and IJsselsteijn, 2006). For instance, such devices have shown promising results in promoting sociability and attentiveness among toddlers (Tanaka et al., 2007) and schoolchildren (Nakanishi et al., 2016). In addition, such systems provide a tremendous opportunity to fill the physical interaction gap in our daily life telecommunication means (e.g., cellphones, Skype, etc.). This is due to their potential for physical embodiment of the intended action/communication and hence fulfilling our inherent needs for physical contact and touch. Research suggests that such mediated communications show capacity to reduce anxiety (Yamazaki et al., 2016). Moreover, the acceptability of these systems as embodied communication media by the senior citizens (Yamazaki et al., 2014) encourage further investigation of their deployment in healthcare facilities (Wada et al., 2004, 2005a,b; Wada and Shibata, 2006).

**Figure 1:**
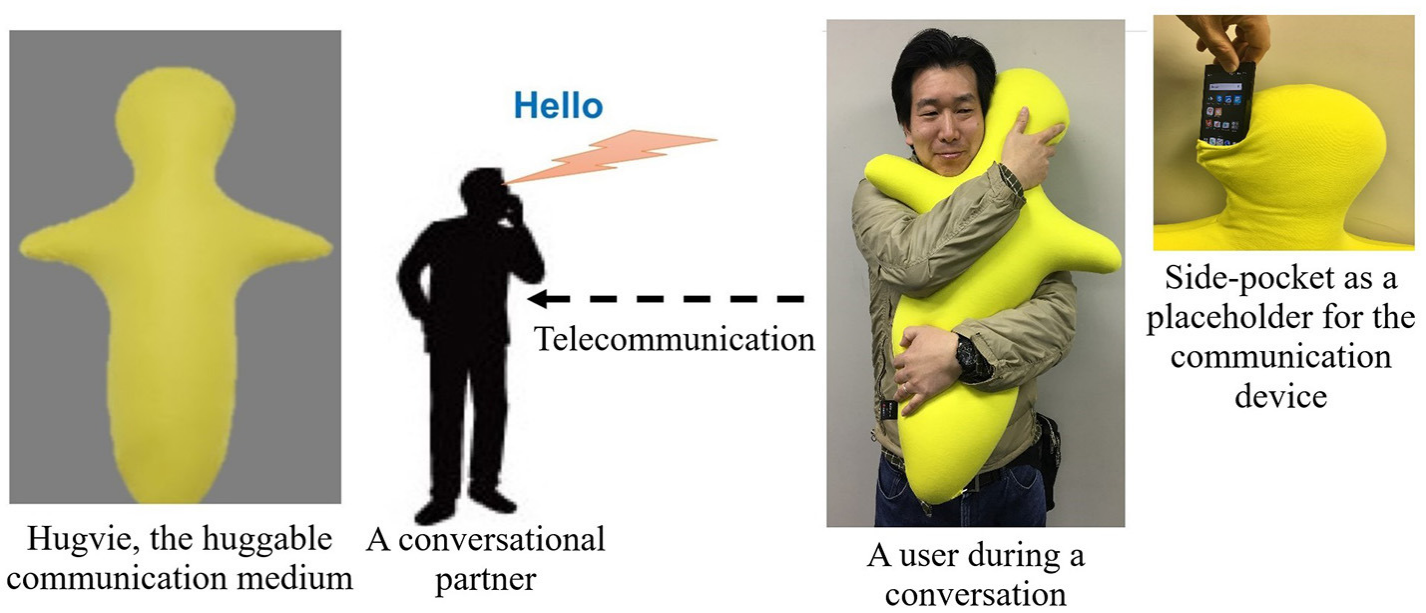
An experimenter demonstrates the use of Hugvie, a huggable telecommunication medium.

Despite these comprehensive studies on the psychological and the behavioral effects of these systems on human subjects, there is a paucity of research on the extent of their physiological impacts. Our earlier empirical result using the huggable communication medium, Hugvie^TM^ (Minato et al., 2013) (please refer to Figure 1), suggests that its use positively influences the endocrine system of human participants (Sumioka et al., 2013). More specifically, the introduction of such a medium during the course of tele-communication results in the reduction of the cortisol level in participants.

However, it is unclear how such a medium affect our brain activity.

In this article, we investigated the effect of the mediated communication through Hugvie based on comparative analysis of the EEG data of human subjects in the presence and the absence of this mediated communication device. In doing so, we were interested in ascertaining answers to following two research questions:

- RQ1. Does such a medium induce any significant difference on the brain activity of the human subjects during a course of communication?
- RQ2. Is such a difference, if any, persistent independent of the content of the communication?

In order to address these research questions, we conducted two series of experiments, involving two groups of participants. We investigated the answer to RQ1 in the first experiment (between-group design) in which participants listened to two different 12-min-long recorded stories either through our huggable communication medium, Hugvie, or a Bluetooth speaker. This allowed for a quantitative analysis of the effect of the presence of our physically embodied medium on brain activity of human subjects during verbal communication. On the other hand, we designed the second experimental setting (within-subject setting) to seek answer to RQ2. In this experimental setting, we communicated the same recorded story through Hugvie twice. In addition, the second experimental setting differed from the first experiment in that it consisted of two resting periods of 1-min duration each. In one resting period, participants rested while holding Hugvie and the other they rested without holding Hugvie. As a result, we were able to investigate whether the potential induced effect by our huggable communication medium, if any, persisted independent of content of communicated content.

We sought answers to these questions via analyses of the global field potential (GFP) (Lehmann and Skrandies, 1980) of the EEG time series of human subjects. GFP is defined as the standard deviation across multiple channels as a function of time. It is a root mean square measure that quantifies the spatial potential field sampled over the scalp. A peak of GFP reflects a maximum of the total underlying brain activity that contributes to the surface potential field (Lehmann and Skrandies, 1980; Haenschel et al., 2000). Therefore, choice of GFP enabled us to evaluate the effect of a physically embodied communication medium on the overall brain activity of human subjects, regardless of the spatial location(s) of the brain region(s) responding to this medium. In other words, it allowed for comparative analyses of such an effect in terms of overall residual change in power while discarding its underlying spatial distribution.

Research on effect of anesthetic substances on state of consciousness of human subjects indicates a significant reduction in relative occurrences of ordinal patterns in EEG time series of human subjects (Silva et al., 2010). In particular, application of permutation entropy (PE) (Bandt and Pompe, 2002) is shown to distinguish between the awake state and unconsciousness at ≈0.4 (Olofsen et al., 2008). Moreover, use of PE in study and analysis of mindfulness and mediation implies its significant reduction during meditative state (Vysata et al., 2014). Taken together, these results suggest the potential of PE for determination of degree of relaxation (from meditative state of mind to total unconsciousness under the influence of an anesthetic substance). Therefore, we used PE of GFP-transformed EEG time series of brain activity of participants in our study as a measure to investigate the potential relaxing effect of Hugvie.

Although PE provides a robust measure for analysis of the overall change in dynamics of EEG time series of brain activity of human subjects, it is unable to quantify the temporal changes in such a dynamics. Determination of such temporal changes in EEG time series of brain activity is of crucial importance, considering its strong correlation with productivity of such cognitive functions as attention and language processing (Goldberger et al., 2002; Takahashi et al., 2009; Manor et al., 2013). Interestingly, an increase in complexity of brain activity reflects the information content of physiological systems which, in turn, has a direct correspondence to variational information of their exhibited activity (Costa et al., 2005a). More importantly, such a variability in brain activity inherently differs from randomness and noisy signals (McDonough and Nashiro, 2014). In this regards, multiscale entropy (MSE) (Goldberger et al., 2000; Costa et al., 2002) presents a robust tool for analysis and quantification of temporal variability of brain activity of human subjects (Garrett et al., 2013). Therefore, we computed MSE of GFP-transformed EEG time series of the participants to analyze the degree of complexity of the brain activity of our participants in response to the communicated stories, thereby realizing the potential influence of Hugvie on dynamics of the brain activity (and subsequently its information content in response to narrated stories) of these individuals.

We expected that the communicated content that is mediated through a device which activates the sense of hug and touch in individuals results in a more pronounced positive influence at the brain activity level, drawing relevant correspondence between the results of our analyses and the findings reported in cognitive neuroscience research (Haenschel et al., 2000; Knyazev, 2011; Sarnthein et al., 2011).

## 2. Methods

### 2.1. Experimental setup

We conducted two series of experiments, referred to as *first experimental setting* and *second experimental setting* hereafter. Their descriptions are as follows.

#### 2.1.1. First experimental setting

It involved two sessions of storytelling. In each session, the stories (recorded audio files) were communicated either through Bluetooth speaker (No-Hugvie condition) or through the same Bluetooth speaker that was placed in the head-side pocket of our huggable telecommunication medium, Hugive (please see Figure 1). It is worth noting that prior to the experiment we received confirmation from Miyuki Giken Co., Ltd, Tokyo, Japan, that bluetooth does not interfere with EEG recording. We instructed our participants to hug this medium throughout the storytelling sessions. We randomly counterbalanced the order of these two sessions to eliminate the potential detrimental effect of the preceding session on the session that follows (e.g., over/under-excitement and/or fatigue). Moreover, we chose “The Fall of Freddie the Leaf” by Leo F. Buscaglia and “The Rabbit Who Wants to Fall Asleep: A New Way of Getting Children to Sleep” by Carl-Johan Forssn Ehrlin as our first and the second stories. Each session lasts for 12 min. We collected EEG data through eight channels (i.e., Fp2, Fz, Cz, F7, T7, T8, CP5, CP6), following 10–20 international standard system. Once the device was properly mounted, we collected 1 min worth of data during the resting period. The first storytelling session started immediately after data collection for resting state data acquisition was complete.This was followed by asking our participants to fill questionnaires regarding the sense of copresence and social presence induced by the remote storyteller (whether through Hugvie or otherwise). We adapted our questions on self-reported sense of copresence and social presence from Nowak and Biocca (2003). We continued with the second storytelling session, following the exact same protocol as in first session. We advised our subjects to stand still, as much as possible, during the recordings.

#### 2.1.2. Second experimental setting

We solely used the first story from the first experimental setting along with two resting phases (one with and another without Hugvie) in this experiment to evaluate the persisting effect of Hugvie. We communicated the selected story with the participants through Hugvie twice, one for each session. The remainder of the experimental setting followed the exact same protocol as in first experimental setting. It is worthy of note that the two resting periods allowed us to investigate the effect of our huggable medium irrespective of the content of the communication.

We conducted the first and the second experiments with two different groups of participants for each of these experimental settings. The first experimental setting helped test the hypothesis that a huggable communication medium induces a significant difference on the brain activity of its users. On the other hand, we designed the second experimental setting to test if such a difference persisted independent of the content of the narrative by the storyteller.

### 2.2. Subjects

Sixteen (eight females, eight males, 20–27 years old, MEAN = 22.13, SD = 1.54) and 19 (five females, 14 males, 20–35 years old, MEAN = 22.40, SD = 4.53) young adults, with normal hearing took part in the first and the second experimental settings. All participants were right-handed, were free of neurological and psychiatric disorders, and had no history of hearing impairment. Prior to the data collection, we received approval (approval code: 16-601-1) from the ethical committee at the Advanced Telecommunications Research Institute International (ATR), Kyoto, Japan. All subjects gave written informed consent in accordance with the Declaration of Helsinki. Subjects were seated in a reclining easy chair in a sound-attenuated and electrically shielded testing chamber, with instructions to fully relax while their eyes closed.

### 2.3. EEG recording

An EEG Cap with eight electrodes arranged in accordance with the international 10–20 system was fitted on the scalp of the subjects. We used an eight-channel wireless EEG system with dry active electrodes (AP108, Polymate Mini, Miyuki Giken Co., Ltd, Tokyo, Japan). The impedances for channels were kept below 100kΩ at the beginning of the measurements. The right earlobe was used as a reference. The EEG was recorded at a sampling rate of 500 Hz.

### 2.4. EEG data processing

First, we excluded channel Fp2 from further processing and analyses due to excessive noise in its recorded EEG in all participants. For the remainder of channels, we applied a notch filter at 60.0 Hz frequency on our raw EEG signals to remove the effect of the base power. Next, we applied a bandpass filter with the low- and high-pass 0.0 and 50.0 Hz, respectively. Furthermore, we removed the motion artifacts from our signals through application of independent component analysis (ICA). We used the open-source Matlab-based FastICA package for this purpose (Hyvärinen et al., 2001).

After preprocessing the signals, we computed the global field potential (GFP) (Lehmann and Skrandies, 1980) of these eight channels, per participant, to carry out our statistical analyses. GFP is defined as the standard deviation across multiple channels as a function of time. It is a root mean square measure that quantifies the spatial potential field sampled over the scalp. A peak of GFP reflects a maximum of the total underlying brain activity that contributes to the surface potential field (Lehmann and Skrandies, 1980; Haenschel et al., 2000). We used the GFP associated with the EEG of the individuals to extract the following frequency bands during our analysis: delta (0–4 Hz), theta (4–8 Hz), alpha (8–12 Hz), beta(12–30 Hz), and gamma (30–50 Hz). It is worth noting that our analyses showed non-significant difference between the use of reference-free and average-reference GFP. Therefore, we utilized the reference-free GFP in our analyses.

### 2.5. Statistical analyses

Our analyses are based on *a priori* hypothesis that presence of Hugvie results in a positive effect at the EEG-level of brain activity of the individual participants. Result of the Kolmogorov–Smirnov test implied that our data was not normally distributed. Therefore, we applied Wilcoxon rank sum test to investigate the difference among the EEG frequency bands of the individuals in the presence and the absence of Hugvie, thereby eliminating any prior assumption on the distribution of the EEG data of our participants.

In addition, we calculated the permutation entropy (PE) (Bandt and Pompe, 2002) of the GFP of the EEG time series of the participants. The PE describes the order relations between the values of a time series. Furthermore, its computation is fast, simple, and robust with respect to the noise. Although its normalized value varies within [0, 1], its minimum practical boundary is ≈0.4 (i.e., the state of unconscious in an anesthetized patient) (Olofsen et al., 2008). PE has been successfully applied in a variety of biomedical and neuroscientific applications ranging from computation of the depth of the anesthetic drug effect (Olofsen et al., 2008; Silva et al., 2010) to the detection of the epilepsy and the epileptic seizure (Cao et al., 2004; Veisi et al., 2007; Mammone et al., 2015). In particular, we utilized this measure to estimate the level of relaxation with respect to the overall power of the EEG signal of the participants due to its application in the detection of the anesthetic effect (Zanin et al., 2012). We applied the Wilcoxon rank sum test while analyzing the PE values of these time series.

Moreover, we compared the complexity of the time series data of our participants using multiscale entropy (MSE) (Costa et al., 2002). MSE finds numerous applications in a number of biosignal analyses ranging from assessment of EEG dynamical complexity in Alzheimer's disease (Mizuno et al., 2010) and epileptic vs. healthy EEG discrimination (Gao et al., 2015) to classification of surface electromyography (EMG) of neuromuscular disorders (Istenic et al., 2010). We applied MSE on the EEG time series of our participants to analyze the presence of any significant difference in dynamics of the pattern of their brain activity over the multiple time scales (Goldberger et al., 2000; Costa et al., 2005a). We used pattern length *m* = 2, similarity criterion *r* = 0.15, and range of 1–20 for the scale factor, as suggested in Goldberger et al. (2000). We utilized the grand average of the MSE values of all the participants (i.e., average of the MSE sequences of length 20, corresponding to range of 1–20 for the scale factor Goldberger et al., 2000) to conduct the Wilcoxon rank sum test.

Considering the configuration of our EEG channels (i.e., Fp2, Fz, Cz, F7, T7, T8, CP5, CP6) and after exclusion of Fp2 (due to excessive noise, as explained in section 2.4) from further analyses, F7 was the only channel that was not hemispherically paired. We excluded F7 and observed that our results were unaffected. Therefore, we decided to report our results on full set of EEG channels (with the exception of Fp2) in this article.

For all cases of Wilcoxon rank sum tests (i.e., power, frequencies, PE, and MSE), we report $r=\frac{W}{\sqrt{N}}$, as suggested by Tomczak and Romczak (2014), where *W* is the Wilcoxon statistics and *N* represent the sample size.

## 3. Results

**Figure 2:**
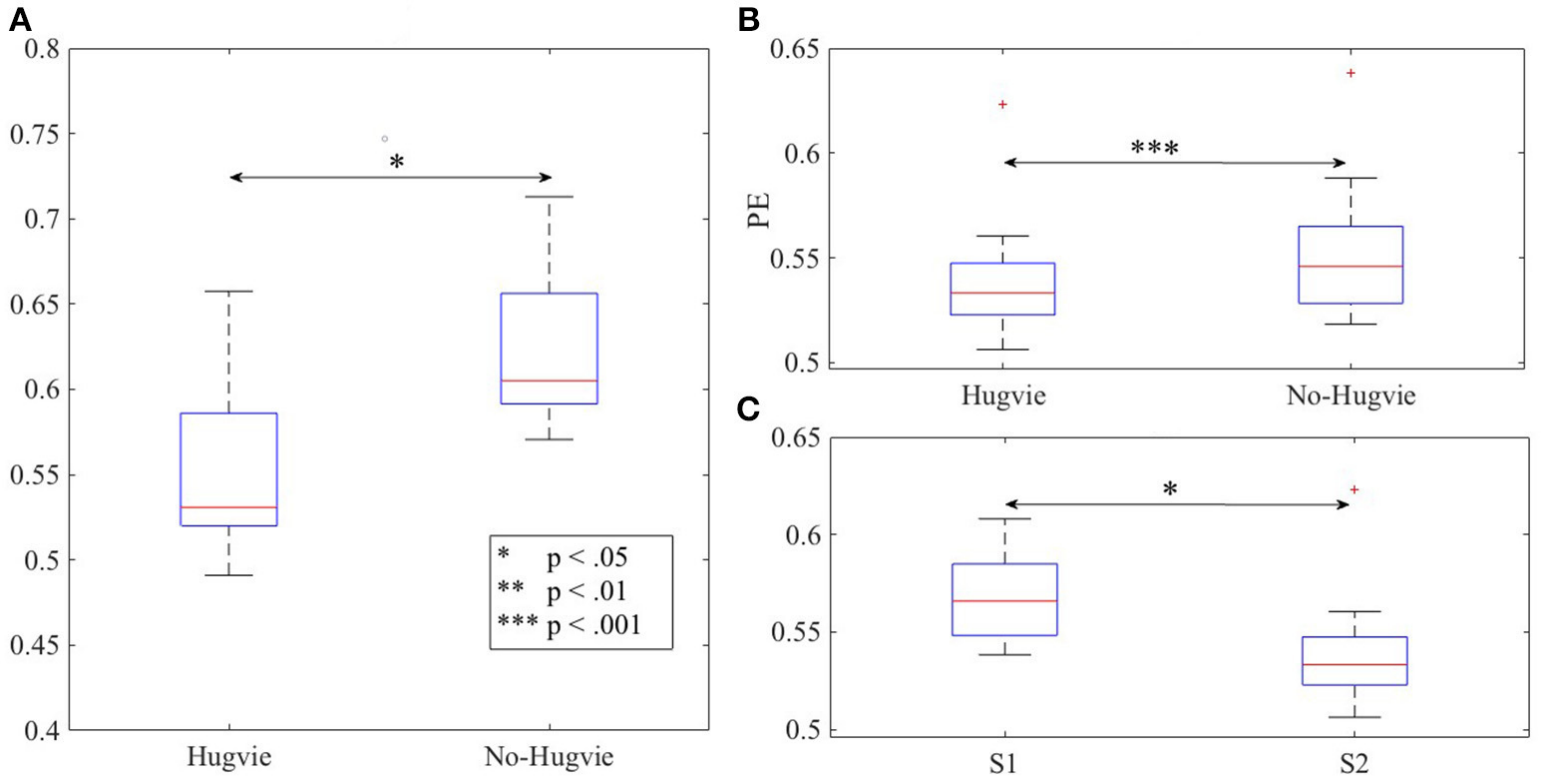
Magnitude of permutation entropies of the EEG time series of the participants **(A)** first experimental setting **(B)** second experimental setting during rest (i.e., with and without Hugvie) **(C)** second experimental setting during first and second storytelling sessions. ^*^p < 0.05; ^**^p < 0.01 ^***^p < 0.001.

As we expected, presence of Hugvie (H) induced significant difference on the brain activity of the participants while listening to the narrated stories. In the first experimental setting, presence of Hugvie resulted in reduction of the EEG power of participants as compared with the No-Hugvie (NH) group [*p* < 0.001, *W*_(15)_ = 324.31, *r* = 83.74], and W(.) is the value of Wilcoxon test statistics. This observation was further supported by the permutation entropy (PE, Figure 2A) indexes of these groups [*p* < 0.05, *W*_(15)_ = 2.05, *r* = 0.53]. Table 1, “Overall Power” and “PE” column entries, summarize these results.

**Table 1:** First Experimental Setting: Wilcoxon rank sum *p*-value, test statistics, and effect size (*r* = $\frac{W}{\sqrt{N}}$ Tomczak and Romczak, 2014) along with mean (M) and standard deviation (SD) for overall power, permutation entropy (PE), and multi-scale entropy (MSE) in Hugvie (H) vs. No-Hugvie (NH) scenarios.

	***p*<**	**W**	***r***	**M_*H*_**	**SD_*H*_**	**M_*NH*_**	**SD_*NH*_**
Overall Power	0.001	324.31	83.74	−110.13	12.61	−82.62	14.42
PE	0.05	2.05	0.53	0.56	0.03	0.61	0.03
MSE	0.001	4.02	1.04	0.42	0.14	0.21	0.08

**Figure 3:**
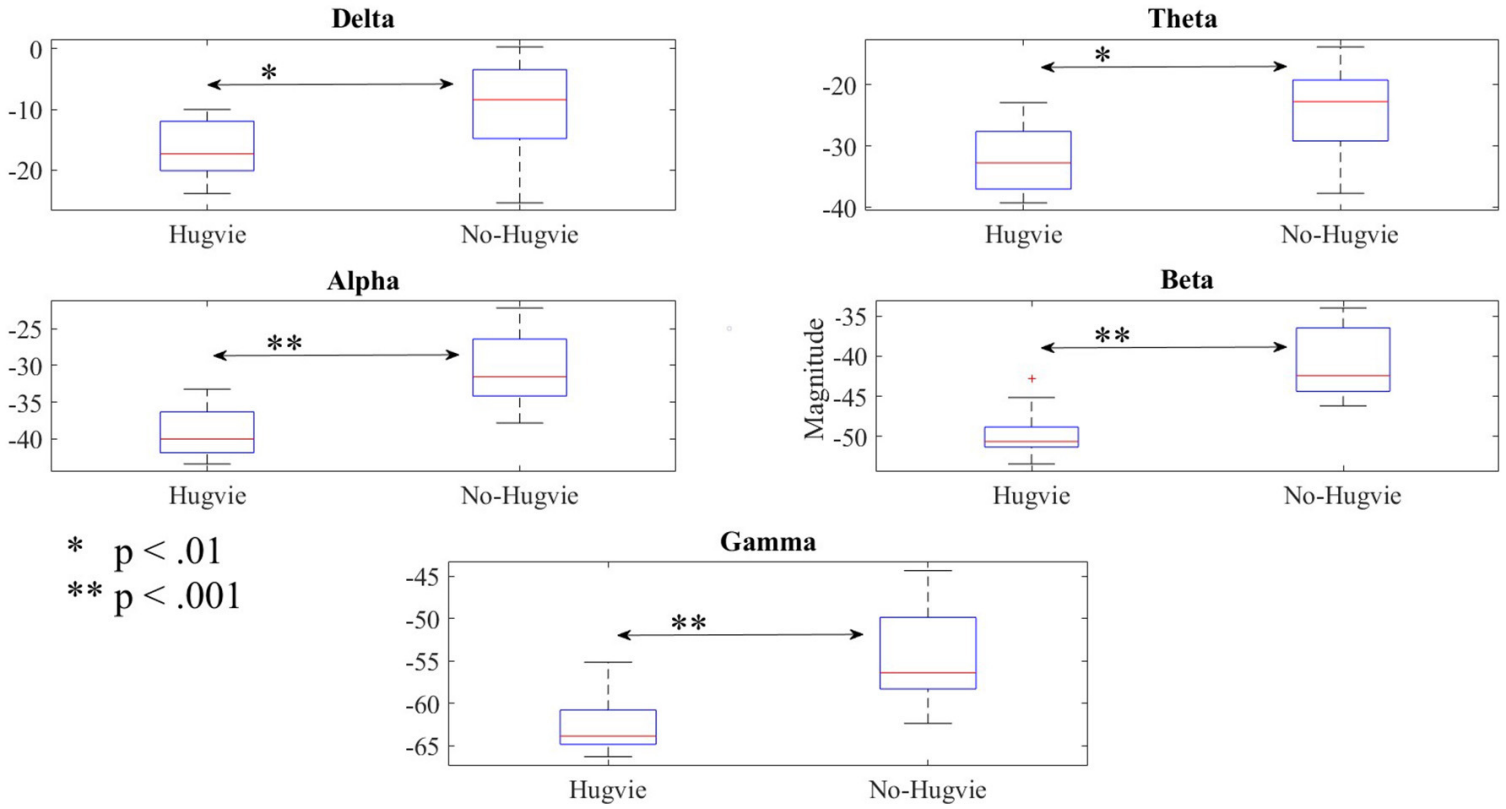
Reduction of power due to presence of Hugvie in different frequency bands of the EEG time series of participants in the first experimental setting.

Figure 3 shows the effect of Hugvie on power in different frequency bands. Presence of Hugvie evoked significant decrease in beta and gamma frequency bands [beta: *p* < 0.001, *W*_(15)_ = 4.47, *r* = 1.15, and gamma: *p* < 0.001, *W*_(15)_ = 3.91, *r* = 1.01]. Moreover, alpha, delta, and theta frequencies significantly decreased in its presence [alpha: *p* < 0.001, *W*_(15)_ = 4.43, *r* = 1.14, delta: *p* < 0.01, *W*_(15)_ = 2.54, *r* = 0.66, theta: *p* < 0.01, *W*_(15)_ = 2.96, *r* = 0.76]. Table 2 summarizes these results.

**Table 2:** First Experimental Setting: Wilcoxon rank sum *p*-value, test statistics, and effect size (*r* = $\frac{W}{\sqrt{N}}$ Tomczak and Romczak, 2014) along with mean (M) and standard deviation (SD) for delta (δ), theta (θ), alpha (α), beta (β), and gamma (γ) in Hugvie (H) vs. No-Hugvie (NH) scenarios.

	***p*<**	**W**	***r***	**M_*H*_**	**SD_*H*_**	**M_*NH*_**	**SD_*NH*_**
δ	0.01	2.54	0.66	−16.40	4.47	−9.76	7.89
θ	0.01	2.96	0.76	−32.07	5.37	−24.33	6.87
α	0.001	4.43	1.14	−39.45	3.12	−30.53	4.55
β	0.001	4.47	1.15	−49.82	2.72	−40.81	4.28
γ	0.001	3.91	1.01	−62.59	3.26	−54.58	5.33

**Figure 4:**
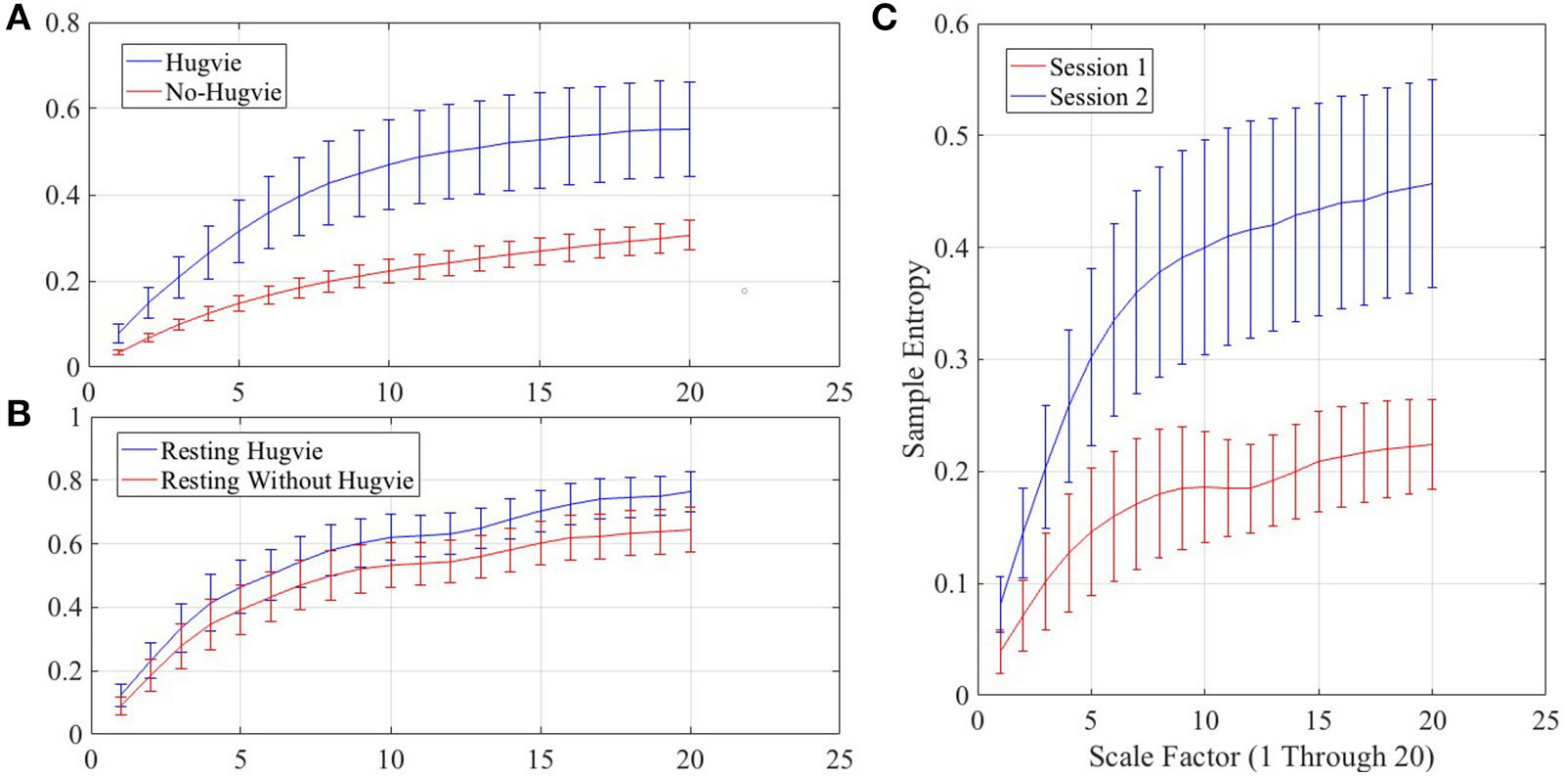
Grand averages of the multiscale entropy (MSE) of the EEG time series of the participants.**(A)** first experimental setting **(B)** second experimental setting while resting with and without Hugvie **(C)** second experimental setting during the sessions one and two. We used pattern length *m* = 2, similarity criterion *r* = 0.15, and range of 1–20 for the scale factor, as suggested in (Goldberger et al., 2000).

**Figure 5:**
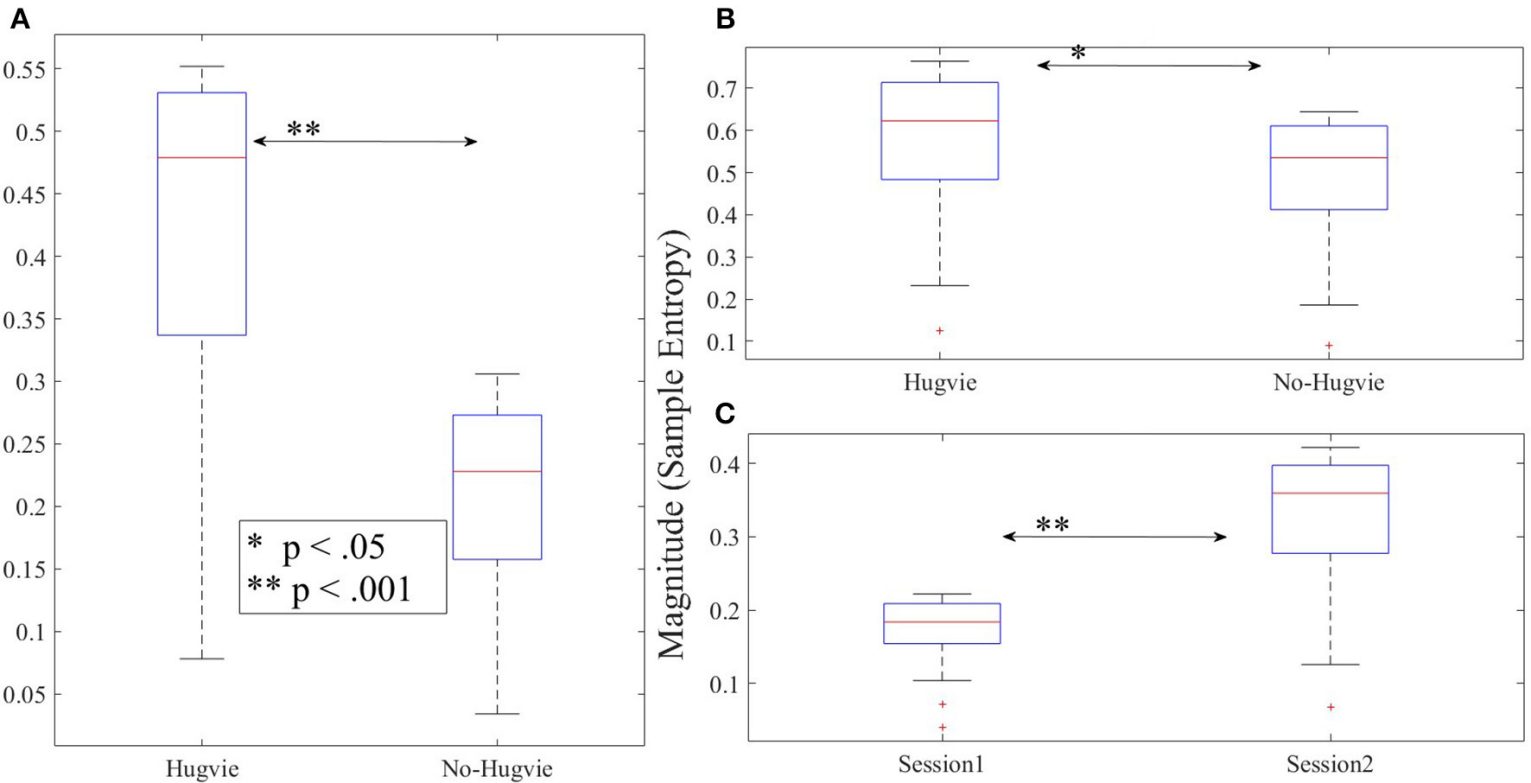
Magnitude of sample entropies as computed by mutli-scale entropy (MSE) of the EEG time series of the participants for scales 1–20 **(A)** first experimental setting **(B)** second experimental setting during rest (i.e., with and without Hugvie) **(C)** second experimental setting during first and second storytelling sessions.

Figure 4A shows the grand average of MSE values for Hugvie and No-Hugvie settings. Analysis of the MSE values of these time series (i.e., Figure 5A) revealed a significant difference between the dynamical pattern of the brain activity of the participants in the presence and the absence of Hugvie [*p* < 0.001, W_(15)_ = 4.02, *r* = 1.04]. Table 1, “MSE” column entry, summarizes these results.

In the second experimental setting, we observed a significant reduced power between the two story sessions [*p* < 0.001, *W*_(18)_ = 184.45, *r* = 43.48]. This observation was supported by significant reduction of PEs, shown in Figure 2C, between these sessions [*p* < 0.02 *W*_(18)_ = 2.30, *r* = 0.54]. Column entries “Overall Power” and “PE” in Table 3 summarize these results.

**Table 3:** Second Experimental Setting: Wilcoxon rank sum *p*-value, test statistics, and effect size (*r* = $\frac{W}{\sqrt{N}}$ Tomczak and Romczak, 2014) along with mean (M) and standard deviation (SD) for overall power, permutation entropy (PE), and multi-scale entropy (MSE) during session1 (S1) and session2 (S2) of storytelling.

	***p*<**	**W**	***r***	**M_*S*1_**	**SD_*S*1_**	**M_*S*2_**	**SD_*S*2_**
Overall Power	0.001	184.45	43.48	−106.17	16.16	−114.52	15.02
PE	0.02	2.30	0.54	0.57	0.02	0.54	0.02
MSE	0.001	4.32	1.02	0.49	0.17	0.57	0.18

Figure 4C shows the grand average of MSE values of these two sessions. Analysis of the MSE values of these time series (i.e., Figure 5C) implied that the temporal complexity of the brain activity of these individuals exhibited an incremental pattern of activity [*p* < 0.001, *W*_(18)_ = 4.32, *r* = 1.02]. Table 1, column entry “MSE,” summarizes these results.

Interestingly, differences between the frequency bands of these two sessions were non-significant [Delta: *p* = 0.33 and *W*_(18)_ = 0.96, *r* = 0.23, Theta: *p* = 0.24 and *W*_(18)_ = 1.17, *r* = 0.28, Alpha: *p* = 0.14 and *W*_(18)_ = 1.46, *r* = 0.34, Beta: *p* = 0.16 and *W*_(18)_ = 1.14, *r* = 0.27, Gamma: *p* = 0.23 and *W*_(18)_ = 1.20, *r* = 0.28].

Table 4 summarizes the overall power and PE values of the participants during resting with (H) and without (NH) Hugvie. Presence of Hugvie induced the same pattern of reduction of power in the resting periods [*p* < 0.001, *W*_(18)_ = 23.61, *r* = 5.56]. Moreover, the PEs (i.e., Figure 2B) of these resting periods supported this significant reduced power in presence of Hugvie (*p* < 0.001, *W*_(18)_ = 3.40, *r* = 0.80].

**Table 4:** Second Experimental Setting: Wilcoxon rank sum *p*-value, test statistics, and effect size (*r* = $\frac{W}{\sqrt{N}}$ Tomczak and Romczak, 2014) along with mean (M) and standard deviation (SD) for overall power, permutation entropy (PE), and multi-scale entropy (MSE) during resting with (H) and without (H) Hugvie.

	***p*<**	**W**	***r***	**M_*H*_**	**SD_*H*_**	**M_*NH*_**	**SD_*NH*_**
Overall Power	0.001	23.61	5.56	−85.05	10.61	−84.26	10.12
PE	0.001	3.40	0.80	0.54	0.02	0.56	0.04
MSE	0.05	2.00	0.47	0.57	0.18	0.49	0.17

Figure 4B shows the grand average of MSE values of these two resting periods. We observed a significant difference between the MSE values of the time series of the participants during the resting with and without Hugvie, where its presence induced a higher complexity in the pattern of brain activity of these individuals (Figure 5B, *p* < 0.05, *W*_(18)_ = 2.00, *r* = 0.47). Column entry “MSE” in Table 4 summarizes these results.

**Figure 6:**
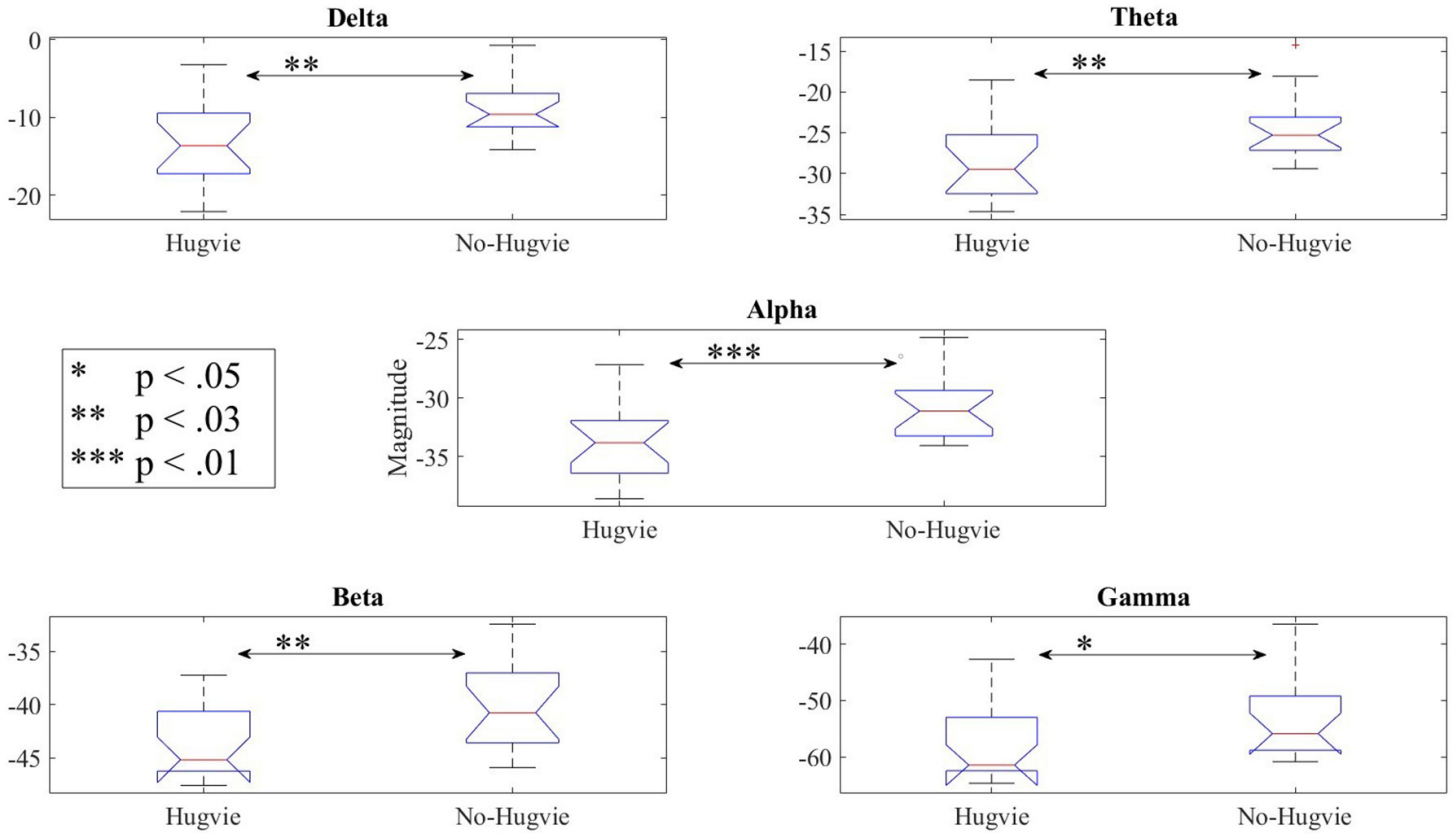
Reduction of power during resting in all frequency bands in the presence of the huggable communication medium and irrespective of communication content.

Lastly, our result indicated significant differences between the frequency bands of resting (i.e., Table 5) with and without Hugvie [Delta: *p* < 03, *W*_(18)_ = 2.31, *r* = 0.54, Theta: *p* < 03, *W*_(18)_ = 2.34, *r* = 0.55, Alpha: *p* < 0.01, *W*_(18)_ = 2.76, *r* = 0.65, Beta: *p* < 0.03, *W*_(18)_ = 2.51, *r* = 0.59, Gamma: *p* < 0.05, *W*_(18)_ = 2.17, *r* = 0.51]. Figure 6 illustrates these results.

**Table 5:** Second Experimental Setting: Wilcoxon rank sum *p*-value, test statistics, and effect size (*r* = $\frac{W}{\sqrt{N}}$ Tomczak and Romczak, 2014) along with mean (M) and standard deviation (SD) for delta (δ), theta (θ), alpha (α), beta (β), and gamma (γ) during resting with (H) and without (H) Hugvie.

	***p*<**	**W**	***r***	**M_*H*_**	**SD_*H*_**	**M_*NH*_**	**SD_*NH*_**
δ	0.03	2.31	0.54	−13.68	4.54	−9.82	3.94
θ	0.03	2.34	0.55	−30.24	3.78	−26.43	3.08
α	0.01	2.76	0.65	−33.88	3.43	−30.23	3.28
β	0.03	2.51	0.59	−44.15	2.69	−41.76	2.31
γ	0.05	2.17	0.51	−57.55	3.97	−54.69	1.82

## 4. Discussion

In this study we investigated whether a physically embodied communication medium induces any significant difference on the brain activity of human subjects during a course of verbal communication. Furthermore, we examined if such a potentially induced difference persisted independent of the communication content. We expected that communication through a medium whose physical embodiment brings about the sense of hug to induce patterns of brain activity that supports the characteristics that are implicit in our research questions.

Results of our analyses verified this expectation. Precisely, we observed that the presence of our embodied communication medium, Hugvie, resulted in a significant reduction of power in brain activity of participants, as compared with the absence of Hugvie, in the first experimental setting. This observation that was further supported by significant reduction of power in different frequency bands of the EEG time series of these participants implied that the use of Hugvie, in fact, induced a significant difference on the brain activity of the human subjects during their course of communication (i.e., RQ1). Additionally, we observed that the power in EEG time series of these participants significantly reduced as we moved from the first to the second session of the storytelling in the second experimental setting while the reduction of the magnitude of power in different frequencies of these time series remained non-significant between these two storytelling sessions. These results implied that the induced differences in the presence of our physically embodied communication medium, Hugvie, in fact persisted independent of the communication content (i.e., RQ2). This is due to the fact that we used the same story content during the two sessions of the second experimental setting.

Remarkably, our results attributed these induced significant differences primarily to the act of hugging. This is due to the observation that the effect of Hugvie during the resting periods in second experimental setting remained in full accord with our results during the first experiment. This result was of great importance due to two reasons. First and foremost, these resting periods, unlike the storytelling sessions in both experimental settings, did not include any communication, thereby discarding the communicated content as a decisive factor in our results. Secondly, these resting periods were substantially shorter than storytelling sessions (i.e., one- in contrast with 12-min-long), thereby discarding the duration of the interaction as an essential factor. Therefore, and with regards to aforementioned factors (i.e., physical embodiment, communication content, and duration of interaction), it is plausible to primarily attribute the induced reduction of overall power (with a very large effect size in all experimental settings and conditions) in EEG time series of the participants to physical embodiment of Hugvie. The attribution of observed reduction of power to the act of hugging in our experiments finds evidence in psychological studies that suggest the direct influence of behavior and posture on formation of emotional perception (Riskind and Gotay, 1982; Aron et al., 1997; Schubert and Koole, 2009). However, it is crucial to point out the potential that some other aspects of a physically embodied medium may impose on these results. For instance, it is necessary to investigate the effect of such qualities as warmth, texture, and softness of a physically embodied medium can bear on our results, given the reports on brain activation in response to tactile effect (Gallace and Spence, 2012) and touch (Field, 2010). Moreover, research suggests that mirror system of human brain responds to actions that are performed by human and robotic agents alike (Gazzola et al., 2007). This implies the importance of determining the effect of morphological aspect of this physically embodied medium (e.g., human-likeness and anthropomorphic design vs. a neutrally designed medium) on these result. Therefore, future research is necessary to determine the utility of these inherent properties of physically embodied media.

An interesting observation with regards to aforementioned results pertains to the reduced PE values of EEG time series of brain activity of the participants while listening to narrated stories in our experimental settings. Research findings on awake state in conjunction with administration of anesthetic substances as well as state of mindfulness during meditation associate the reduced PE to loss of consciousness (Olofsen et al., 2008; Silva et al., 2010) and relaxed state of mind (Vysata et al., 2014), respectively. Considering these findings, it is plausible to interpret the reduced PE values of the EEG time series of the participants in the first and the second experimental settings along with its decrease in resting with Hugvie setting (as opposed to resting without Hugvie) as an indication of induced feeling of relaxed mood by this physically embodied communication medium. Further support to this line of reasoning comes from the observed reduction of power in alpha and beta frequencies that is in accord with the effect of massage therapy on stress relief (Shagass, 1972; Klimesch et al., 1999; Diego et al., 2004) along with the reduction of beta during a certain meditation practices (Hinterberger et al., 2014). This interpretations are also evident in positive effect of Hugvie on reduced cortisol level of human subjects (Sumioka et al., 2013).

Kerlin et al. (2010) use a multi-source auditory paradigm (referred to as “cocktail party”) to show that increased power in theta frequency pertains to the attended talker (i.e., choice of auditory source) while alpha rhythm increases in response to the direction of auditory attention (i.e., its spatial information). These results are further observed in response to auditory stimuli along with their correspondences with known effect of visual stimuli on these frequency bands by Banrejee et al. (2011). Furthermore, increased power in delta is reported to reflect the absorption state in hypnotic state (Rainville et al., 2002), decreased vigilance and attention (Paus et al., 1997), as well as slow wave sleep (Kajimura et al., 1999). It is also worthy of note that despite the potential correspondence between increased power in alpha and attention (Klimesch, 2012), such an incremental pattern of activity is unwarranted in case of tasks with high demand on the frontal and parietal cortex, attentive processing of external stimuli, as well as intentional mental operation with high cognitive load (Laufs et al., 2003). Moreover, Gevins et al. (1997) indicate a decrease of power in alpha in response to working memory load, thereby indicating its inverse proportionality to the allocated cortical resources while performing spatial and verbal tasks. Therefore, considering the active listening nature of our experimental settings (i.e., attended auditory stimuli), strong correspondence between such tasks and (medial)PFC (Baddeley, 2003; Mar, 2011), along with the fact that our experimental settings incorporate single auditory source with the direction of whose is fixed (i.e., recorded stories through a single, fixed speaker), it is plausible to attribute observed overall reduction of power in these frequency bands in our participants along with accompanying decrease in PE to relaxing effect of the presence of our physically embodied medium.

An important observation that requires further explanation is the decreased power in beta and gamma frequencies. Research findings associate the increase of power in these frequencies with selective attention and vigilance (Olufsen et al., 2003; Reynolds and Chelazzi, 2004; Börgers et al., 2005; Howells et al., 2012). Additionally, increase in gamma power is associated with excessive load on working memory (WM) (Howard et al., 2003; Fries et al., 2007). In this respect, the physical embodiment of communication medium appears to induce a relaxed mode that decreases the degree of vigilance and the demand on cognitive resources for processing of the auditory stimuli by WM. This interpretation is also in accord with observed decrease of beta power during meditation (Gómez et al., 1998; Amihai and Kozhevnikov, 2014). Therefore, it is plausible to interpret the reduced power in beta and gamma frequencies in presence of physically embodied medium as an indication of ease in following the course of narrated stories.

Further evidence on the potential positive effect of this physically embodied communication medium was provided through the result of the analyses of the MSEs of the time series of the participants in the first and the second experimental settings. More specifically, the analysis of the complexity of these time series in their different degrees of temporal resolution (as reflected in varying scaling factor of MSE) revealed that the presence of Hugvie resulted in a higher complexity of the brain activity of these participants during the first experimental setting. Similarly, our analysis suggested the same significant increase in complexity of the brain activity of the participants during the resting period in presence of Hugvie, as compared to their values in its absence, in the second experimental setting. More importantly, we observed a significant increase of MSEs as we moved from session1 to session2 of the storytelling of the second experimental setting. Although it is plausible to expect a reduction of MSE as a complementary result to observed reduction of PE values in response to induced feeling of relaxed mood, such an interpretation is unwarranted due to two main reasons. Firstly and as noted by a number of research findings (Zhang, 1991; Costa et al., 2002, 2005a), deterministic as well as full random signals are not really complex. This implies that change in pattern of activity of brain activity, as reflected in measures such as PE and MSE, cannot be interpreted in terms of reduction and/or increase in randomness of the given time series but the change in their degree of variability in response to stimuli. Secondly, increase in signal variability, as quantified by MSE, is associated with a number of cognitive functions as attention and language processing (Goldberger et al., 2002; Takahashi et al., 2009; Manor et al., 2013), learning (Heisz et al., 2012), as well as reduced behavioral variability and maturation of the brain in various stages of aging (McIntosh et al., 2008, 2013; Garrett et al., 2013). Therefore, it is plausible to interpret the increased MSEs of EEG time series of brain activity of participants in Hugvie setting as an indication of their involvement and attention to communicated contents.

A wide range of developmental disorders is associated with the increased relative power of the low EEG frequencies, including the delta and the theta bands (Knyazev, 2011; Moretti, 2015). Moreover, these low frequencies show increased activity in attention-deficit/hyperactivity disorder (ADHD), predominantly the theta, but also the delta in children with ADHD compared to the control group (Barry et al., 2003). Additionally, these activities are observed in children with Down syndrome (Babiloni et al., 2009), people with schizophrenia (Fehr et al., 2001), depression (Gatt et al., 2008), adults with anxiety disorder (Gauthier et al., 2009), and neurogenic pain (Sarnthein et al., 2011), as well as victims of torture and physical abuse (Ray et al., 2006) (please refer to Knyazev, 2011 for a exhaustive review of the literature on this topic). In addition, excess of spectral power in low frequency bands is associated with the disorders in learning and attention in children (Chabot et al., 2001; Knyazev, 2011). Reduction of power in delta and theta frequencies in presence of Hugvie, as indicated in the first experimental setting as well as resting periods in the second experimental setting (please refer to Figures 3, 6) suggests the use of such physically embodied huggable telecommunication media as a complementary step in treatment of patients struggling with such mental and emotional anomalies. This suggestion is further strengthened through observation of the reduction of the overall EEG power of the brain activity of our participants along with their corresponding PE values. For instance, Yamazaki et al. (2016) show that these embodied tele-communication media help reduce the anxiety of the participants. In addition, Sumioka et al. (2013) show that communication while hugging such media help reduce blood cortisol level. Our results suggest that these influences are in fact present at the brain activity level.

Taken together, our results implied the significant effect of hug, as opposed to no-hug condition, on brain activity of human subjects during verbal communication. Moreover, they implied that such an effect is primarily due to the hug and is unaffected by the communicated content and/or duration of interaction. These results that, to the best of our knowledge, represent the first direct comparative analyses of effect of hug on brain activity of human subjects during the course of telecommunication (in comparison with no-hug condition) are in line with our earlier findings on effect of a physically embodied communication medium on reduction of cortisol level in human subjects (Sumioka et al., 2013). These results that are also in accord with neuroscientific findings on positive effect of physical interaction (as suggested by massage therapy) on stress relief (Shagass, 1972; Klimesch et al., 1999; Diego et al., 2004), induced feeling of relaxation (as suggested by reduced PE values of brain activity of our participants) during mediation (Hinterberger et al., 2014; Vysata et al., 2014), as well as potential increase in involvement and attentiveness of our participants [due to increase in MSE of their brain activity, as observed in such cognitive functions as attention and language processing (Goldberger et al., 2002; Takahashi et al., 2009; Manor et al., 2013) and learning (Heisz et al., 2012)] suggest that the use of these media are potential complementary tools in treatment of developmental disorders, children with ADHD, attentiveness of schoolchildren and early child development, as well as scenarios where intimate physical interaction over distance is desirable (e.g., distance-parenting). However, we strongly believe that further investigation of these effects on larger group of human subjects along with richer number of EEG channels (in particular in orbitofrontal, parietal, and temporal areas, given the more pronounced effect of auditory and tactile effects on these regions; Field, 2010; Gallace and Spence, 2012; Müller and Lindenberger, 2012) is necessary to realize the true potential that such media can offer to the solution concept of more physically enhanced tele-communication scenarios.

One the limitation of present study is the absence of a control medium in which the communication is routed without any physical contact between the medium and the participants. Therefore, it is necessary to include this setting to further assert the observed effect of hug on brain activity of human subjects during verbal telecommunication. Moreover, It is of utmost importance to determine the utility of physical properties of such physically embodied media (e.g., texture, warmth, softness, and morphology), given the tactile effect on brain activity (Field, 2010; Gallace and Spence, 2012). In particular, it is crucial to clarify whether human-likeness of the shape of Hugvie bears any positive influence on observed significant differences in brain activity of human subjects (Gazzola et al., 2007) or these effects can be solely explained in light of physical embodiment of this communication medium. It is also crucial to compare and analyze these results in contrast with the effect of other media (e.g., cell phone, TV, etc.) on the brain activity to determine the utility of the physical embodiment of the medium that we use in this study.

## Ethics statement

This study was carried out in accordance with the recommendations of the ethical committee of Advanced Telecommunications Research Institute International (ATR) with written informed consent from all subjects. All subjects gave written informed consent in accordance with the Declaration of Helsinki. The protocol was approved by the ATR ethical committee (approval code:16-601-1).

## Author contributions

HS conceived the experiments and reviewed the manuscript. JN conducted the experiments. SK analyzed the results and prepared the manuscript. As the head of Hiroshi Ishiguro Laboratories (HIL), HI oversees the entire activity of all research teams and themes, ensuring the soundness of all proposals, quality of results, and their validity.

### Conflict of interest statement

The authors declare that the research was conducted in the absence of any commercial or financial relationships that could be construed as a potential conflict of interest.

## References

[B1] Amihai I. Kozhevnikov M. (2014). Arousal vs. relaxation: a comparison of the neurophysiological and cognitive correlates of vajrayana and theravada meditative practices. PLoS ONE 9:102990. 10.1371/journal.pone.0102990 25051268 PMC4106862

[B2] Aron A. Melinat E. Vallone R. Bator R. (1997). The experimental generation of interpersonal closeness: a procedure and some preliminary findings. Pers. Soc. Psychol. 23, 363–377. 10.1177/0146167297234003

[B3] Babiloni C. Albertini G. Onorati P. Vecchio F. Buffo P. Sar M.. (2009). Inter-hemispheric functional coupling of eyes-closed resting *EEG* rhythms in adolescents with down syndrome. Clin. Neurophisiol. 120, 1619–1627. 10.1016/j.clinph.2009.06.017 19643663

[B4] Baddeley A. (2003). Working memory: looking back and looking forward. Nat. Rev. Neurosci. 4, 829–839. 10.1038/nrn1201 14523382

[B5] Bandt C. Pompe B. (2002). Permutation entropy: a natural complexity measure for time series. Phys. Rev. Lett. 88:174102. 10.1103/PhysRevLett.88.174102 12005759

[B6] Banrejee S. Snyder A. Molholm S. Foxe J. (2011). Oscillatory alpha-band mechanism and the deployment of spatial attention to anticipated auditory and visual target locations: supramodal or sensory-specific control mechanism. J. Neurosci. 31, 9923–9932. 10.1523/JNEUROSCI.4660-10.2011 21734284 PMC3343376

[B7] Barry R. J. Clarke A. R. Johnstone S. J. (2003). A review of electrophysiology in attention-deficit/hyperactivity disorder: I. qualitative and quantitative electroencephalography. Clin. Neurophysiol. 114, 171–183. 10.1016/S1388-2457(02)00362-0 12559224

[B8] Blackmore S. J. Bristow D. Bird G. Frith C. Ward J. (2005). Somatosensory activations during the observation of touch and a case of vision-touch synaesthesia. Brain 128, 1571–1583. 10.1093/brain/awh500 15817510

[B9] Börgers C. Epstein S. Kopell N. (2005). Background gamma rhythmicity and attention in cortical local circuits: a computational study. Proc. Natl. Acad. Sci. U.S.A. 102, 7002–7007. 10.1073/pnas.0502366102 15870189 PMC1100794

[B10] Cao Y. Tung W.-W. Gao J. Protopopescu V. Hively L. (2004). Detecting dynamical changes in time series using the permutation entropy. Phys. Rev. E 70:046217. 10.1103/PhysRevE.70.046217 15600505

[B11] Chabot R. J. di Michele F. Prichep L. John E. (2001). The clinical role of computerized *EEG* in the evaluation and treatment of learning and attention disorders in children and adolescents. Neuropsychiatry Clin. Neurosci. 13, 171–186. 10.1176/appi.neuropsych.13.2.171 11449024

[B12] Chatel-Goldman J. Congedo M. Jutten C. Schwartz J. (2014). Touch increases autonomic coupling between romantic partners. Front. Behav. Neurosci. 8:95. 10.3389/fnbeh.2014.00095 24734009 PMC3973922

[B13] Gómez C. M. Vazquez M. E. V. Lopez-Mendoza D. Cardoso M. (1998). Frequency analysis of the EEG during spatial selective attention. Int. J. Neurosci. 95, 17–32. 10.3109/00207459809000646 9845013

[B14] Costa M. Goldberger A. Peng C.-K. (2002). Multiscale entropy analysis of complex physiologic time series. Phys. Rev. Lett. 89:068102. 10.1103/PhysRevLett.89.068102 12190613

[B15] Costa M. Goldberger A. Peng C.-K. (2005a). Multiscale analysis of biological signals. Phys. Rev. E 71:021906. 10.1103/PhysRevE.71.021906 15783351

[B16] Diego M. A. Field T. Sanders C. Hernandez-Reif M. (2004). Massage therapy of moderate and light pressure and vibrator effects on *EEG* and heart rate. Int. J. Neurosci. 114, 31–45. 10.1080/00207450490249446 14660065

[B17] Fehr T. Kissler J. Moratti S. Wienbruch C. Rockstroh B. Elbert T. (2001). Source distribution of neuromagnetic slow waves and *MEG*-delta activity in schizophrenic patients. Biol. Psychiatry 50, 108–116. 10.1016/S0006-3223(01)01122-2 11526991

[B18] Field T. (2010). Touch for socioemotional and physical well-being: a review. Dev. Rev. 30, 367–383. 10.1016/j.dr.2011.01.001

[B19] Field T. Diego M. Schanberg S. Kuhn C. (2005). Cortisol decreases and serotonin and dopamine increase following massage therapy. Int. J. Neurosci. 115, 1397–1413. 10.1080/00207450590956459 16162447

[B20] Fries P. Nikolic D. Singer W. (2007). The gamma cycle. Trends Neurosci. 30, 309–316. 10.1016/j.tins.2007.05.005 17555828

[B21] Gallace A. Spence C. (2010). The science of interpersonal touch: an overview. Neuroscience 34, 246–259. 10.1016/j.neubiorev.2008.10.004 18992276

[B22] Gallace A. Spence C. (2012). The cognitive and neural correlates of tactile memory. Psychol. Bull. 135:380. 10.1037/a0015325 19379022

[B23] Gao J. Hu J. Liu F. Cao Y. (2015). Multiscale entropy analysis of biological signals: a fundamental bi-scaling law. Front. Comput. Neurosci. 9:64. 10.3389/fncom.2015.00064 26082711 PMC4451367

[B24] Garrett D. D. Samanez-Larkin G. MacDonald S. Lindenberger U. McIntosh A. Grady C. (2013). Moment-to-moment brain signal variability: a next frontier in human brain mapping? Neurosci. Biobehav. Rev. 37, 610–624. 10.1016/j.neubiorev.2013.02.015 23458776 PMC3732213

[B25] Gatt J. M. Kuan S. Dobson-Stone C. Paul R. Joffe R. Kemp A.. (2008). Association between bdnf val66met polymorphism and trait depression is mediated via resting *EEG* alpha band activity. Biol. Psychol. 79, 275–284. 10.1016/j.biopsycho.2008.07.004 18721847

[B26] Gauthier A. K. Chevrette T. Bouvier H. Godbout R. (2009). Evening vs. morning wake *EEG* activity in adolescents with anxiety disorders. J. Anxiety Disord. 23, 112–117. 10.1016/j.janxdis.2008.04.005 18555659

[B27] Gazzola V. Rizzolatti G. Wicker B. Keysers C. (2007). The anthropomorphic brain: the mirror neuron system responds to human and robotic actions. Neuroimage 35, 1674–1684. 10.1016/j.neuroimage.2007.02.003 17395490

[B28] Gevins A. Smith M. McEvoy L. Yu D. (1997). High-resolution *EEG* mapping of cortical activation related to working memory: effects of task difficulty, type of processing, and practice. Cereb. Cortex 7, 374–385. 10.1093/cercor/7.4.374 9177767

[B29] Goldberger A. L. Amaral L. Glass L. Hausdorff J. Ivanov P. Mark P.. (2000). Physiobank, physiotoolkit, and physionet: components of a new research resource for complex physiologic signals. Circulation 101, e215–e220. 10.1161/01.CIR.101.23.e215 10851218

[B30] Goldberger A. L. Amaral L. Hausdorff J. Ivanov P. Peng C. Stanely H. (2002). Fractal dynamics in physiology: alterations with disease and aging. Proc. Natl. Acad. Sci. U.S.A. 99, 2466–2472. 10.1073/pnas.012579499 11875196 PMC128562

[B31] Haans A. IJsselsteijn W. (2006). Mediated social touch: a review of current research and future directions. Virtual Reality 9, 149–159. 10.1007/s10055-005-0014-2

[B32] Haenschel C. Baldeweg T. Croft R. J. Whittington M. Gruzelier J. (2000). Gamma and beta frequency oscillations in response to novel auditory stimuli: a comparison of human electroencephalogram (*EEG*) data with *in vitro* models. Proc. Natl. Acad. Sci. U.S.A. 97, 7645–765. 10.1073/pnas.120162397 10852953 PMC16599

[B33] Heisz J. J. Shedden J. M. McIntosh A. R. (2012). Relating brain signal variability to knowledge representation. Neuroimage 63, 1384–1392. 10.1016/j.neuroimage.2012.08.018 22906786

[B34] Hinterberger T. Schmidt S. Kamei T. Walach H. (2014). Decreased electrophysiological activity represents the conscious state of emptiness in meditation. Front. Psychol. 5:99. 10.3389/fpsyg.2014.00099 24596562 PMC3925830

[B35] Howard M. W. Rizzuto D. Caplan J. Madsen J. Lisman J. Aschenbrenner-Scheibe R.. (2003). Gamma oscillations correlate with working memory load in humans. Cereb. Cortex 13, 1369–1374. 10.1093/cercor/bhg084 14615302

[B36] Howells F. M. Ives-Deliperi V. Horn N. Stein D. (2012). Mindfulness based cognitive therapy improves frontal control in bipolar disorder: a pilot eeg study. BMC Psychiatry 12:15. 10.1186/1471-244X-12-15 22375965 PMC3305658

[B37] Hyvärinen A. Karhunen J. Oja E. (2001). Independent Component Analysis. New York, NY: John Wiley & Sons.

[B38] Istenic R. Kaplanis P. Pattichis C. Zazula D. (2010). Multiscale entropy-based approach to automated surface EMG classification of neuromuscular disorders. Med. Biol. Eng. Comput. 48, 773–781. 10.1007/s11517-010-0629-7 20490940

[B39] Kajimura N. Uchiyama M. Takayama Y. Uchida S. Uema T. Kato M.. (1999). Activity of midbrain reticular formation and neocortex during the progression of human non-rapid eye movement sleep. J. Neurosci. 19, 10065–10073. 10.1523/JNEUROSCI.19-22-10065.1999 10559414 PMC6782956

[B40] Kerlin J. Shahin A. Miller L. (2010). Attentional gain control of ongoing cortical speech representation in a “cocktail party.” J. Neurosci. 30, 620–628. 10.1523/JNEUROSCI.3631-09.2010 20071526 PMC2832933

[B41] Klimesch W. (2012). Alpha-band oscillations, attention, and controlled access to stored information. Trends Cogn. Sci. 16, 606–617. 10.1016/j.tics.2012.10.007 23141428 PMC3507158

[B42] Klimesch W. Doppelmayer M. Russegger H. Pachinger T. Shwaiger J. (1999). Induced alpha band power changes in human *EEG* and attention. Adolescence 34, 529–534. 9572588 10.1016/s0304-3940(98)00122-0

[B43] Knapp M. Hall J. Horgan T. (2012). Nonverbal Communication in Human Interaction. Boston, MA: Cengage Learning.

[B44] Knyazev G. (2011). *EEG* delta oscillations as a correlate of basic homeostatic and motivational processes. Neurosci. Biobehav. Rev. 36, 677–695. 10.1016/j.neubiorev.2011.10.002 22020231

[B45] Kutner J. S. Smith M. Corbin L. Hemphill L. Benton K. Mellis B.. (2008). Massage therapy versus simple touch to improve pain and mood in patients with advanced cancer: a randomized trial. Ann. Intern. Med. 149, 369–379. 10.7326/0003-4819-149-6-200809160-00003 18794556 PMC2631433

[B46] Laufs H. Krakow K. Sterzer P. Eger E. Beyerle A. Salek-Haddadi A.. (2003). Electroencephalographic signatures of attentional and cognitive default modes in spontaneous brain activity fluctuations at rest. Proc. Natl. Acad. Sci. U.S.A. 100, 11053–11058. 10.1073/pnas.1831638100 12958209 PMC196925

[B47] Lehmann D. Skrandies W. (1980). Reference-free identification of components of checkerboard-evoked multichannel potential fields. Electroencephalogr. Clin. Neurophysiol. 48, 609–621. 10.1016/0013-4694(80)90419-8 6155251

[B48] Löken L. S. Wessberg J. Marrison I. McGlone F. Olausson H. (2012). Coding of pleasant touch by unmyelinated afferents in humans. Nat. Neurosci. 12, 547–548. 10.1038/nn.2312 19363489

[B49] Mammone N. Duun-Henriksen J. Kjaer T. Morabito F. (2015). Differentiating interictal and ictal states in childhood absence epilepsy through permutation renyi entropy. Entropy 17, 4627–4643. 10.3390/e17074627

[B50] Manor B. Lipsitz L. A. (2013). Physiologic complexity and aging: implications for physical function and rehabilitation. Progr. Neuro-Psychopharmacol. Biol. Psychiatry 45, 287–293. 10.1016/j.pnpbp.2012.08.020 22985940 PMC3568237

[B51] Mar R. (2011). The neural bases of social cognition and story comprehension. Annu. Rev. Psychol. 62, 103–134. 10.1146/annurev-psych-120709-145406 21126178

[B52] McDonough I. M. Nashiro K. (2014). Network complexity as ameasure of information processing across resting-state networks: evidence from the human connectome project. Front. Hum. Neurosci. 8:409. 10.3389/fnhum.2014.00409 24959130 PMC4051265

[B53] McIntosh A. R. Kovacevic N. Itier R. (2008). Increased brain signal variability accompanies lower behavioral variability in development. PLoS Comput. Biol. 4:e1000106. 10.1371/journal.pcbi.1000106 18604265 PMC2429973

[B54] McIntosh A. R. Vakorin V. Kovacevic N. Wang H. Diaconescu A. Protzner A. (2013). Spatiotemporal dependency of age-related changes in brain signal variability. Cereb. Cortex 24, 1806–1817. 10.1093/cercor/bht030 23395850 PMC4051893

[B55] Minato T. Nishio S. Ishiguro H. (2013). Evoking an affection for communication partner by a robotic communication medium, in Proceedings of the 8^th^ ACM/IEEE International Conference on Human-Robot Interaction D07 (Tokyo), D07.

[B56] Mizuno T. Takahashi T. Cho R. Kikuchi M. Murata T. Takahashi K. (2010). Assessment of *EEG* dynamical complexity in alzheimer's disease using multiscale entropy. Clin. Neurophysiol. 121, 1438–1446. 10.1016/j.clinph.2010.03.025 20400371 PMC2914820

[B57] Moretti D. (2015). Theta and alpha *EEG* frequency interplay in subjects with mild cognitive impairment: evidence from *EEG*, mri, and spect brain modifications. Front. Aging Neurosci. 7:31. 10.3389/fnagi.2015.00031 25926789 PMC4396516

[B58] Müller V. Lindenberger U. (2012). Lifespan differences in nonlinear dynamics during rest and auditory oddball performance. Dev. Sci. 15, 540–556. 10.1111/j.1467-7687.2012.01153 22709403

[B59] Nakanishi J. Sumioka H. Ishiguro H. (2016). Impact of mediated intimate interaction on education: a huggable communication medium that encourages listening. Front. Psychol. 7:510. 10.3389/fpsyg.2016.00510 27148119 PMC4835693

[B60] Nowak K. Biocca F. (2003). The effect of the agency and anthropomorphism on users' sense of telepresence, copresence, and social presence in virtual environment. Presence 12, 481–494. 10.1162/105474603322761289

[B61] Olofsen E. Sleigh J. Dahan A. (2008). Permutation entropy of the electroencephalogram: a measure of anesthetic drug effect. Br. J. Anaesth. 101, 810–821. 10.1093/bja/aen290 18852113

[B62] Olufsen M. S. Whittington M. Camperi M. Kopell N. (2003). New roles for the gamma rhythm: population tuning and preprocessing for the beta rhythm. J. Comput. Neurosci. 14, 33–54. 10.1023/A:1021124317706 12435923

[B63] Paus T. Zatorre R. Hofle N. Caramanos Z. Gotman J. Petrides M.. (1997). Time-related changes in neural systems underlying attention and arousal during the performance of an auditory vigilance task. J. Cogn. Neurosci. 9, 392–408. 10.1162/jocn.1997.9.3.392 23965014

[B64] Rainville P. Hofbauer R. Bushnell M. Duncan G. Price D. (2002). Hypnosis modulates activity in brain structures involved in the regulation of consciousness. J. Cogn. Neurosci. 14, 887–901. 10.1162/089892902760191117 12191456

[B65] Ray W. J. Odenwald M. Neuner F. Schauer M. Ruf M. Weinbruch C.. (2006). Decoupling neural networks from reality: dissociative experiences in torture victims are reflected in abnormal brain waves in left frontal cortex. Psychol. Sci. 17, 825–829. 10.1111/j.1467-9280.2006.01788.x 17100779

[B66] Reynolds J. H. Chelazzi L. (2004). Attentional modulation of visual processing. Annu. Rev. Neurosci. 27, 611–647. 10.1146/annurev.neuro.26.041002.131039 15217345

[B67] Riskind J. Gotay C. (1982). Physical posture: could it have regulatory or feedback effects on motivation and emotion? Motiv. Emot. 6, 273–298. 10.1007/BF00992249

[B68] Sarnthein J. J. Stern Aufenberg C. Rousson V. Jeanmonod D. (2011). Increased *EEG* power and slowed dominant frequency in patients with neurogenic pain. Brain 129, 55–64. 10.1093/brain/awh631 16183660

[B69] Schubert T. Koole S. (2009). The embodied self: making a fist enhances men's power-related self-conceptions. J. Exp. Soc. Psychol. 45, 828–834. 10.1016/j.jesp.2009.02.003

[B70] Shagass C. (1972). Electrical activity of the brain, Handbook of Psychophysiology, eds Greenfield N. S. Sternbach R. A. (Oxford: Rinehart & Winston), 263–328.

[B71] Shibata T. (2004). An overview of human interactive robots for psychological enrichment. Proc. IEEE 92, 1749–1758. 10.1109/JPROC.2004.835383

[B72] Silva A. Cardoso-Cruz H. Silva F. Galhardo V. Antunes L. (2010). Comparison of anesthetic depth indexes based on thalamocortical local field potentials in rats. Anesthology 112, 355–363. 10.1097/ALN.0b013e3181ca3196 20098138

[B73] Singh H Bauer M. Chowanski W. Sui Y. Atkinson D. Baurley S.. (2014). The brain's response to pleasant touch: an *EEG* investigation of tactile caressing. Front. Hum. Neurosci. 8:893. 10.3389/fnhum.2014.00893 25426047 PMC4226147

[B74] Sumioka H. Nakae A. Kanai R. Ishiguro H. (2013). Huggable communication medium decreases cortisol levels. Scient. Rep. 3:3034. 10.1038/srep03034 24150186 PMC3805974

[B75] Takahashi T. Cho R. Murata T. Mizuno T. Kikuchi M. Mizukami K. (2009). Age-related variation in eeg complexity to photic stimulation: a multiscale entropy analysis. Clin. Neurophysiol. 120, 476–483. 10.1016/j.clinph.2008.12.043 19231279 PMC2880484

[B76] Tanaka F. Cicourel A. Movellan J. (2007). Socialization between toddlers and robots at an early childhood education center. Proc. Natl. Acad. Sci. U.S.A. 104, 17954–17958. 10.1073/pnas.0707769104 17984068 PMC2084278

[B77] Tomczak M. Romczak E. (2014). The need to report effect size estimates revisited. an overview of some recommended measures of effect size. Trends Sport Sci. 1, 19–25.

[B78] Veisi I. Pariz N. Karimpour A. (2007). Fast and robust detection of epilepsy in noisy *EEG* signals using permutation entropy in Proceedings of the 7th IEEE International Conference on Bioinformatics and Bioengineering.

[B79] Vysata O. Schatz M. Kopal J. Burian J. Prochazka A. Jiri K. (2014). Non-linear *EEG* measures in meditation. J. Biomed. Sci. Eng. 7, 731–738. 10.4236/jbise.2014.79072

[B80] Wada K. Saito T. Tanie K. (2004). Effects of robot-assisted activity for elderly people and nurses at a day service center. Proc. IEEE 92, 1780–1788. 10.1109/JPROC.2004.835378

[B81] Wada K. Shibata T. (2006). Robot therapy in a care house - its sociopsychological and physiological effects on the residents, in Proceedings of the IEEE International Conference on Robotics and Automation (Florida, FL), 3966–3971.

[B82] Wada K. Shibata T. Musha T. Kimura S. (2005a). Effects of robot therapy for demented patients evaluated by *EEG*, in IEEE/RSJ International Conference on Intelligent Robots and Systems (IROS) (Las Vegas, NV), 2847–2852.

[B83] Wada K. Shibata T. Saito T. Sakamoto K. Tanie K. (2005b). Psychological and social effects of one year robot assisted activity on elderly people at a health service facility for the aged, in Proceedings of the IEEE International Conference on Robotics and Automation (Barcelona), 2785–2790.

[B84] Yamazaki R. Christensen L. Shov K. Chang C.-C. Damholdt M. Sumioka H.. (2016). Intimacy in phone conversations: anxiety reduction for danish seniors with hugvie. Front. Psychol. 7:537. 10.3389/fpsyg.2016.00537 27148144 PMC4835483

[B85] Yamazaki R. Nishio S. Ishiguro H. Norskov M. Ishiguro N. Balistreri G. (2014). Acceptability of a teleoperated android by senior citizens in danish society. Int. J. Soc. Robot. 6, 429–442. 10.1007/s12369-014-0247-x

[B86] Zanin M. Zunino L. Rosso O. Papo D. (2012). Permutation entropy and its main biomedical and econophysics applications: a review. Entropy 14, 1553–1577. 10.3390/e14081553

[B87] Zhang Y.-C. (1991). Complexity and $\frac{1}{f}$ noise. a phase space approach. J. Phys I EDP 1, 971–977.

